# Non-linear growth in tree ferns, *Dicksonia antarctica* and *Cyathea australis*

**DOI:** 10.1371/journal.pone.0176908

**Published:** 2017-05-11

**Authors:** David P. Blair, Wade Blanchard, Sam C. Banks, David B. Lindenmayer

**Affiliations:** 1 Fenner School of Environment and Society, The Australian National University, Canberra, Australian Capital Territory, Australia; 2 Long-term Ecological Research Network, Fenner School of Environment and Society, The Australian National University, Canberra, Australian Capital Territory, Australia; Universite du Quebec a Chicoutimi, CANADA

## Abstract

Tree ferns are an important structural component of forests in many countries. However, because their regeneration is often unrelated to major disturbances, their age is often difficult to determine. In addition, rates of growth may not be uniform, which further complicates attempts to determine their age. In this study, we measured 5 years of growth of *Cyathea australis* and *Dicksonia antarctica* after a large wildfire in 2009 in south-eastern Australia. We found growth rates of these two species were unaffected by aspect and elevation but slope had a minor effect with *D*. *antarctica* growing 0.3mm faster for each additional degree of slope. Geographic location influenced growth in both species by up to 12 – 14mm/yr. The most consistent factor influencing growth rate, however, was initial height at the time of the 2009 fire; a finding consistent in both species and all geographic locations. For both tree fern species, individuals that were taller at the commencement of the study had greater overall growth for the duration of the study. This effect did not decrease even among the tallest tree ferns in our study (up to 6 metres tall). Overall, *Cyathea australis* averaged 73 (± 22)mm/year of growth (± 1SD), with the rate increasing 5mm/yr per metre of additional height. *Dicksonia antarctica* averaged 33 (± 13)mm/year, increasing by 6mm/yr/m. Growth rates dependent on initial height were unexpected and we discuss possible reasons for this finding. Variable growth rates also suggest that common age estimation methods of dividing height by average growth rate are likely to underestimate the age of short tree ferns, while overestimating the age of tall tree ferns, particularly if they have been subject to a fire.

## Introduction

Tree ferns are found in wet forests worldwide, from tropical regions to cool temperate forests [1, 2]. Tree ferns are generally considered to be slow-growing, long-lived plants that do not require disturbance for reproduction, and as such, become increasingly common in the late successional stage of older forests [3, 4]. Tree ferns often fill important physical and ecological roles. In many forests, including those in Costa Rica, New Zealand and Australia, tree fern trunks host a wide range of epiphytic ferns and bryophytes [5–7], often supporting a greater diversity of such kinds of plants than the other trees in the same forest [8]. Tree ferns influence the presence of other species both positively and negatively, for example, in the forests of south-eastern Australia there are positive relationships between the abundance of tree ferns and the occurrence of arboreal mammals such as the Mountain Brushtail Possum *Trichosurus cunninghami* [9] with these plant species providing both habitat structure and a food source [10], while in southern New Zealand, tree ferns have been found to negatively influence the regeneration success of other trees [11].

In the cool temperate forests of south-eastern Australia where we completed this study, tree ferns are well adapted to the prevailing regime of infrequent but high severity fire [12], resulting in these midstorey species often being older than the overstorey eucalypts, the majority of which are obligate seeders in this investigation [13, 14]. The tree fern species in this study can grow to over ten metres in height [15] and have been estimated to live more than 500 years [13], while other species in Australia have been recorded growing in excess of 15m, including the Norfolk Tree Fern, *Cyathea brownii*, which can grow to 20m [16]. Tree ferns also may be an indicator of past disturbance history given their ability to survive fire, but susceptibility to logging [17–19]. Because tree ferns regularly survive fire but do not require fire to regenerate, their age is not easily determined from the dates of previous major fires. While there have been several studies examining the conditions required for tree ferns to persist, there have been relatively few investigations of the effects of tree fern age, growth rates, or the environmental factors on growth rates, particularly in Australia [12, 13, 20–22].

We studied the short-term growth rates of the two most common species of tree fern occurring in south-eastern Australia, the Rough Tree Fern (*Cyathea australis*) (R. Br.), family Cyatheaceae and Soft Tree Fern (*Dicksonia antarctica*) (Labill), family Dicksoniaceae. The genus *Cyathea* has Pan-Tropical origins [23] with approximately 700 species globally [15] in the tropics, subtropics, and southern temperate zones [2] and includes 11 species in Australia. The genus *Dicksonia* is of Gondwanan origin [23] with 50 species found in south-eastern Australia, New Zealand, south-east Asia and Central and South America. There are three species of *Dicksonia* in Australia [15]. In our study region, the distributions of *D*. *antarctica* and *C*. *australis* overlap, with both found predominantly in cool, wet gullies. *Cyathea australis* is more tolerant of drier micro-climates, and commonly found in the mid to lower elevations where it is warmer and drier and away from streams; *D*. *antarctica* is most often found at mid to higher elevations where annual rainfall is greater, particularly at cold, wet sites close to streams [24].

Tree ferns grow by producing new fronds which extend from the centre of the apical trunk, extending and growing to the outside edge of the trunk. Over a period of 6–12 months, the older fronds gradually deteriorate and die, to be replaced by new layers of fronds with each new layer adding to the overall height of the fern’s trunk. The older fronds eventually drop off and leave the base of the stipe on the trunk. In the study reported here, we quantified the short-term growth rates of *D*. *antarctica* and *C*. *australis* immediately following the Black Saturday wildfires in 2009 in Victoria. Thus, all tree ferns were burnt (with all fronds removed) with trunks of tree ferns blackened to the height they were at the time of the fire. New growth was easily distinguishable in the subsequent years and it was the height of this new growth that we subsequently measured. Previous investigations have measured fern growth following fire [22] and made estimates of maximum age [13] by focussing on the largest individuals, while other studies examined the physiological effects of different temperatures and amounts of light on the photosynthetic abilities of tree ferns [21, 25], although these factors were not then related to resulting changes in growth rate. No previous studies in the wet forests of Victoria, and few investigations internationally [26–29], have measured the growth of tree fern trunks in field conditions, spanning a wide range of heights (and therefore ages) to determine if growth rates of overall trunk height vary with age.

We posed three key questions with related hypotheses: What are the rates of growth of *D*. *antarctica* and *C*. *australis* and do rates of growth vary between these two species? What factors, including environmental variables, influence the rate of growth of *D*. *antarctica* and *C*. *australis*? Finally, from our results: Are we able to devise a simple equation of dividing height by annual growth rate to estimate tree fern age? Given there is no clear consensus within the literature on which species grows faster, and at the leaf scale both have equivalent photosynthetic capabilities [20], we hypothesised that *C*. *australis* would have similar growth rates to *D*. *antarctica* and that growth of both species of tree ferns will be affected by environmental variables. We postulated both species of tree ferns will grow most rapidly at higher elevations. In our study area, elevation is strongly positively correlated with rainfall and temperature. Previous field studies of these two species revealed a lack of effect of seasonal water use efficiency [20], leading us to postulate that rainfall would not be a determining factor for growth in this area (which receives some of the highest and most consistent rainfall in mainland Australia [30, 31]). Temperature has been found to affect tree fern growth in these two species. Lower elevations (our lowest sites were 275m ASL) are subject to periodic very high temperatures during summer, with temperatures in excess of 40°C. While Volkova et al [25] found no change in photosynthetic capacity in *D*. *antartica* with high temperatures (35°C) under shade, when combined with high irradiance, severe photoinhibition was witnessed. On our burned sites, high irradiance was common, thus the higher temperatures at these lower altitudinal sites were expected to reduce growth. Conversely, the lowest temperatures in winter are recorded on our highest sites (up to 985m ASL), with snow falling most winters. Such low temperatures were also found to inhibit photosynthesis [20]. Snow could potentially retard growth by reducing solar interception, however snow does not persist for long periods (usually days at most, not weeks). On balance, we postulated it was most likely the greater reduction in quantum yield, leading to overall lower trunk height growth, would come from the higher temperatures in summer when photosynthetic activity is greatest and is the time of year when the tree ferns have their greatest period of growth [20, 32]. We further postulated tree ferns growing on sites on hotter and drier northerly aspects were likely to grow more slowly than on sites on cooler aspects due to slower growth associated with hotter temperatures [25]. We did not expect geographic location to affect growth. Given most studies on tree ferns in Australia assume a constant growth rate through time [13, 22], we expected to be able to develop a relatively simple growth equation based on annual growth increments, however several international studies on other tree fern species suggest this may not be possible due to variable growth over the life of the tree ferns [26–29].

## Methods

**Fig 1:**
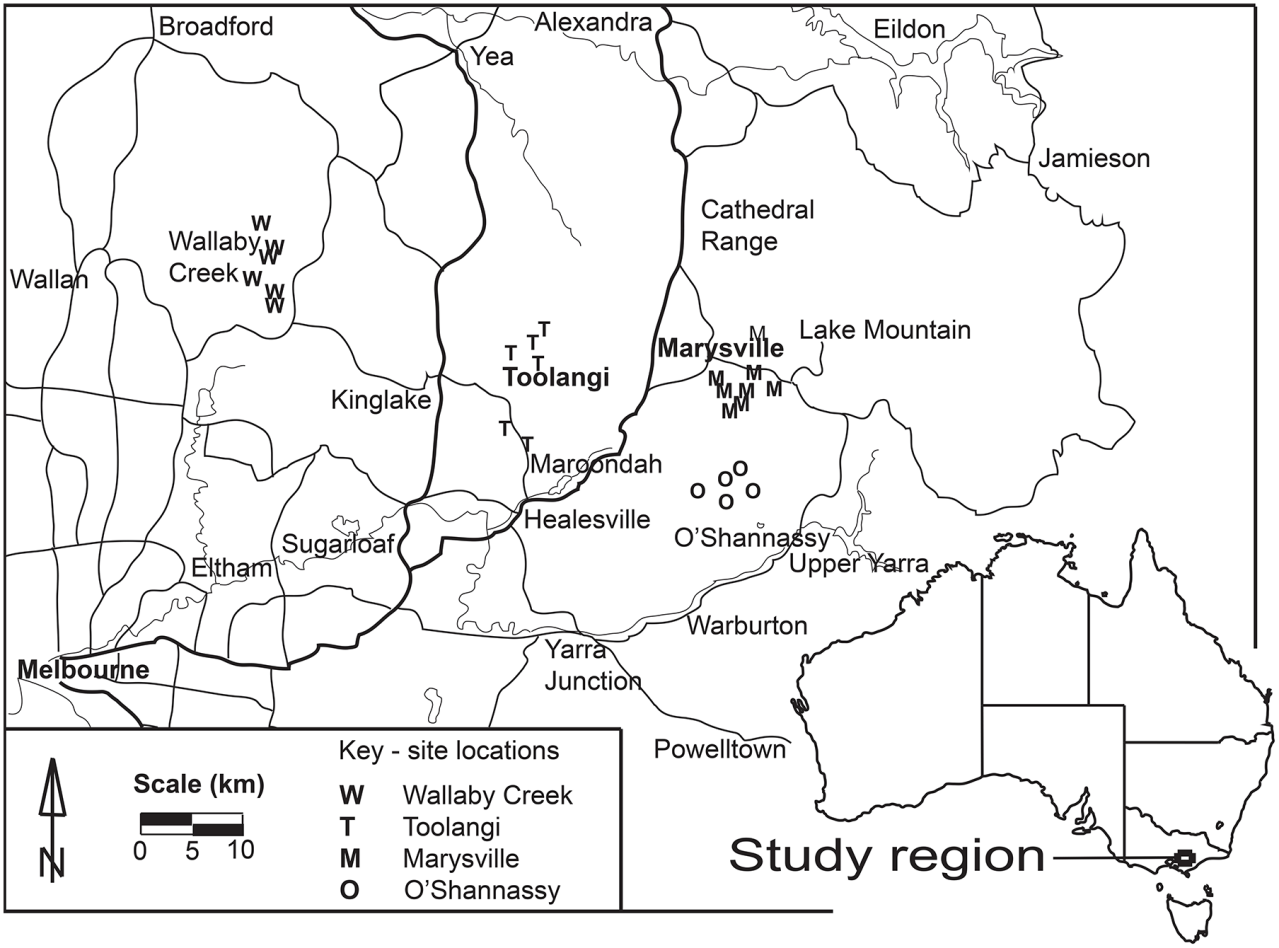
Map of study region showing location of sites.

Our study focused on the forests of the Central Highlands of Victoria, 60-120km east of Melbourne in south eastern Australia (Fig 1).

### Sample areas and fern selection

We measured tree ferns at 25 sites within four separate geographic locations: Marysville State Forest, O’Shannassy water catchment, Toolangi State Forest, and Wallaby Creek water catchment (see Fig 1). At each geographic location, we selected 5–8 sites, and then measured up to ten tree ferns of each species at each site. In total, we measured 163 stems of *Cyathea australis* ranging in pre-fire height from 0.37m to 6.20m and 172 stems of *Dicksonia antarctica*, with heights between 0.28m to 5.03m (Table 1). Each site was selected to allow 10 tree ferns to be measured close to each other (each site having a maximum 25m radius) and with limited variation in slope, aspect and elevation. While there was uniformity of environmental variables *within* sites, we specifically chose sites around each geographic location that would cover a wide range of environmental variables (aspect, slope and altitude) *between* sites. Each site was a minimum of 1 km apart to minimise the potential for spatial depencence to influence our results.

**Table 1:** Summary of attributes for C. australis and D. antarctica at geographic area and site level.

	*C*. *australis*	*D*. *antarctica*	Total for study
**Number of geographic areas**	3	4	4
**Number of sites**	16	17	25
**Number of ferns measured**	163	172	335
**Height range (time of fire)**	0.37 – 6.20m	0.28 – 5.03m	0.28 – 6.20m
**Altitude range**	275m – 975m	395m – 985m	275m – 985m
**Slope range**	0° – 32°	1° – 28°	0° – 32°

The dominant forest types where our sites were located included mixed (eucalypt) species foothill forest dominated by Messmate (*Eucalyptus obliqua*), Peppermints (*E*. *radiata* and *E*. *dives*) and Stringybarks (predominantly *E*. *macrorhyncha*), and at higher elevations, forests dominated by Mountain Ash (*E*. *regnans*) and Alpine Ash (*E*. *delegatensis*). The majority of our sites (21 of our 25 sites), were in the ash forests. Large wildfires dominate the ecology of these forests with intense, stand replacing fires historically occurring on average every 75–150 years [33].

To ensure all tree ferns had had their trunks burnt at the same time (February 2009) and physically blackened uniformly up the trunk (up to 6m tall), we selected sites that had burned at moderate to high severity in the 2009 wildfires. We used fire severity maps from the Victorian Government GIS layer and local knowledge to identify suitable locations. On the Victorian Government fire severity scale, this included areas burned at severity 1–3 (on a scale of 1–6, where 1 is highest severity) and in which the midstorey was fully scorched [34, 35].

At each site, we measured 10 tree ferns of each species unless insufficent tree ferns of either species were able to be found. Eight of the 25 sites supported 10 individuals of both tree fern species. We measured 10 of the tree ferns nearest to the centroid of each site, ensuring that ferns of differing heights were measured. We elected not to measure ferns with multiple trunks, tree ferns with any section of the trunk leaning at an angle of 30° or greater, or ferns that had fallen over and continued to grow. We located the centre of each site away from road edges to reduce potential effects of additional light, temperature differences or water run off.

### Determining growth rates

To determine the growth rates of tree ferns, we located tree ferns with trunks that showed clear delineation between the lower part of the trunk which had been burnt by the February 2009 wildfires and the unburnt upper section of trunk resulting from subsequent growth in the following 5 years, when the ferns were measured between February and April 2014. We measured the overall height of each tree fern using a tape measure, while the new growth was measured using callipers. We then divided the new growth from the last five years (post fire growth) by five to give an overall annual average rate of growth for each individual tree fern.

We took more than two thousand photographs of the study region progressively over the 5 years between the time of the fires to the time of measurement and by looking at these and through field observations, we were able to determine the majority of tree ferns had visible new fronds within 1–4 months of the 2009 fire, indicating growth typically did not appear to have paused due to the fires, despite rare occurrences where some individuals took up to a year after fire to re-sprout.

### Statistical analyses

We used a linear random effects model to explore relationships between annual growth rate and geographic region, elevation, slope, aspect and initial height of the tree fern post 2009 wildfire. Aspect of each site was allocated to the nearest of the eight major compass points (north, north east, east etc.). North, north-west and north-east were collated as ‘northerly aspects’ and analysed against all other aspects combined. This was done to ascertain whether the hotter, drier conditions of the northerly aspects effected tree fern growth. Rainfall within the study area is highly positively correlated with elevation with the summer (November-March) rainfall correlation being 0.910 and winter (April–October) being 0.717. The analysis for both altitude and rainfall resulted in similar findings, therefore we excluded rainfall from subsequent analyses. Site was treated as a random effect to account for potential correlation among the tree ferns at the same site. We used the MCMCglmm package [36] to fit the models in R 3.2.1 [37]. The model parameters are summarized by the posterior mean, 95% credible intervals and Btail, which gives the fraction of the posterior distribution that is to the left or right of zero conditional on whether the posterior mean was greater or less than zero, respectively. Small values of Btail indicated support for non-zero parameter values, that is, posterior distributions that are shifted away from zero.

We did not perform model selection, but rather chose to interpret the full model for each tree fern species. We also performed a diagnostic analysis to assess the underlying assumption of normality and to assess the need to include a site-level random effect. There was very little support for the inclusion of the site-level random effect, and hence our results show the models without this term.

### Ethics statement

Our research required no ethics approvals as we were undertaking non-destructive vegetation measuring without collection of any vegetation samples and we were not studying fauna. Our sites were all in publicly accessible locations on public land controlled by the Victorian Government, and therefore no special permission was required.

## Results

### Overall tree fern growth rates

From our field data, we calculated the overall average growth of *C*. *australis* to be 73 (+/- 22)mm/year of growth (+/- 1 SD) and 33 (+/- 13)mm/year for *D*. *antarctica* (Table 2).

**Table 2:** Summary of growth rates of C. australis and D. antarctica, for all ferns measured on all sites.

	*C*. *australis*	*D*. *antactica*
**Number of tree ferns measured (n)**	163	172
**Average growth (mm/yr)**	73	33
**Standard Deviation (mm/yr)**	22	13
**Maximum growth of an individual fern (mm/yr)**	135	75
**Minimum growth of an individual fern (mm/yr)**	19	7
**Additional growth per m of height (mm)**	5	6

### Growth rates and initial height

For both *C*. *australis* and *D*. *antarctica*, initial height in 2009 was the most significant (Btail <0.001) determinant of growth rates. *Cyathea australis* grew an additional 5mm each year for every additional meter in height the tree fern was at the time of the fire. For *D*. *antarctica*, the increase in growth for taller ferns was even greater with an additional 6mm of additional growth measured each year for each meter taller the ferns were at the time of the fire (Table 2).

### Growth rates and environmental variables

Environmental variables had limited influence on growth rates in *C*. *australis* with no significant effect identified for slope, elevation or aspect. In contrast, *D*. *antarctica* grew more rapidly on steeper slopes at a rate of 0.3mm/year for each additional degree in slope (Btail = 0.010). Elevation and aspect had no influence on the growth rate of *D*. *antarctica* and *C*. *australis*.

### Growth rates and geographic location

**Fig 2:**
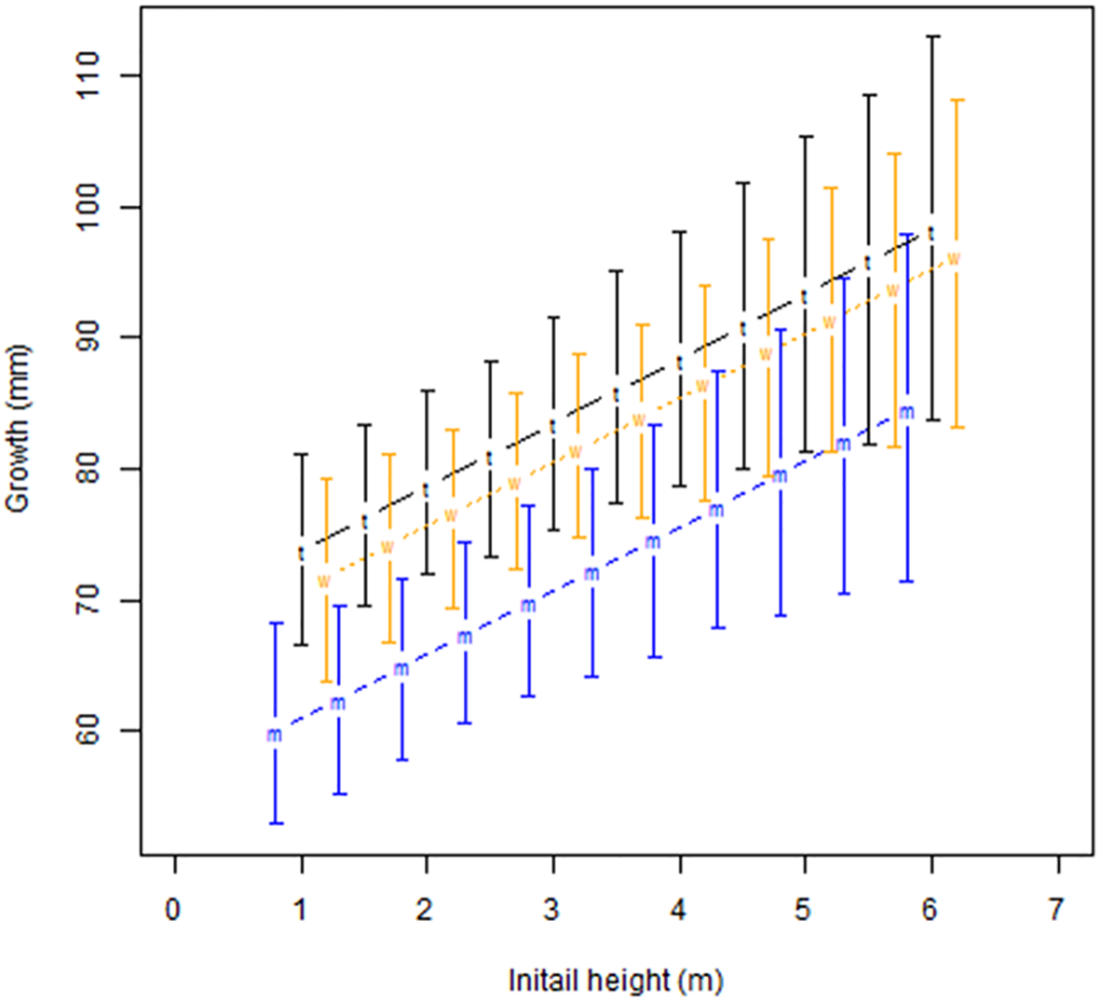
Annual growth of *C*. *australis* by initial height and geographic area (note that height has been slightly offset for each geographic region to improve readability), based on modelled data. m = Marysville State Forest, t = Toolangi State Forest, w = Wallaby Creek water catchment.

Other than initial height in 2009, geographic location had the largest effect on growth rates. *Cyathea australis* grew most rapidly in the Toolangi region, with the growth rate being 13.8mm/yr faster than tree ferns in Marysville (Btail = 0.002). Tree ferns in Wallaby Creek grew 11.7mm/yr faster than in Marysville (Btail = 0.009) (Fig 2). There were insufficient numbers of *C*. *australis* in the O’Shannassy water catchment for statistical analyses of geographic location effects.

**Fig 3:**
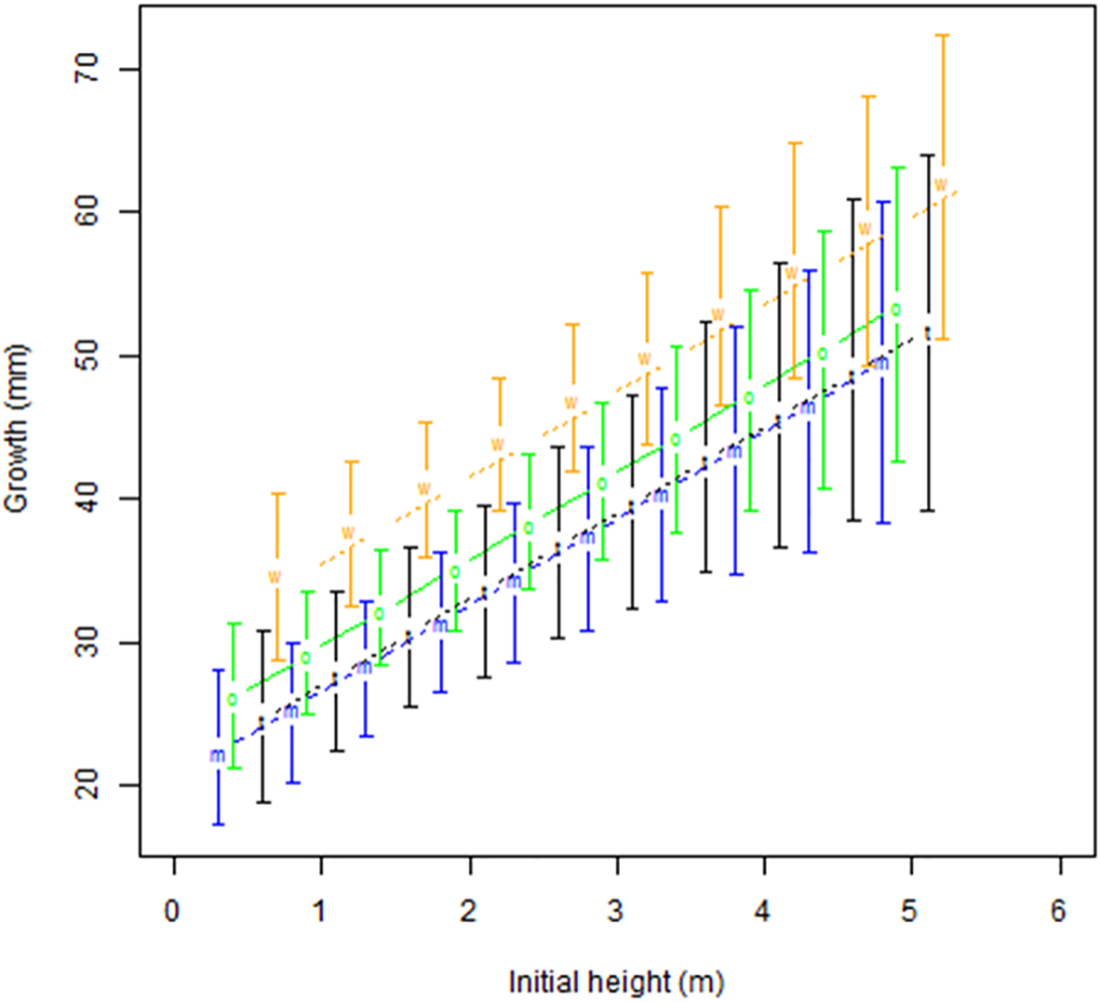
Annual growth of *D*. *antarctica* by initial height and geographic area (note that height has been slightly offset for each geographic region to improve readability), based on modelled data. m = Marysville State Forest, o = O’Shannassy water catchment, t = Toolangi State Forest, w = Wallaby Creek water catchment.

*Dicksonia antarctica* grew most rapidly in the Wallaby Creek water catchment and was 12.5mm/yr faster than in Marysville State Forest (Btail = 0.002), 10.2mm/yr faster than in Toolangi State Forest (Btail = 0.002), and 8.8mm/yr faster than in O’Shannassy water catchment (Btail = 0.004). There was no significant difference in growth rates between the other three geographic locations for this species (Fig 3).

## Discussion

We posed a series of questions relating to the growth of the two most common tree fern species in south-eastern Australia, *D*. *antarctica* and *C*. *australis*, and the factors that influenced their growth. We expected that the environmental variables we measured would influence growth with the more favourable conditions being in areas at higher elevation, where temperatures are lower. Snow falls are common in winter at sites at higher elevation but this was thought to be of little consequence due to its limited persistence and occurrence at the time of year when tree ferns grow the least [32]. At lower elevations, high temperatures and drier conditions throughout the summer are common which are likely to affect growth rates, especially when combined with high irradiance [25]. At the outset of this investigation, we expected growth of both species to be lower on the hotter and drier northern and western aspects compared to cooler southern and eastern aspects. With the forests of the four geographic areas having broadly similar rainfall (long term average between 1194-1393mm per annum for the 4 areas,[38]), we expected similar growth rates across the different geographic locations where other environmental variables such as aspect, elevation and slope were the same.

We found growth rates varied in unexpected ways with initial height at the time of the fire being the strongest determinant of growth rate. In the following sections, we discuss possible explanations for our findings.

### Comparison of tree fern growth rates

Values for the average growth rate of *C*. *australis* and *D*. *antarctica* were broadly within the range of two other earlier studies in south-eastern Australia [13, 22] (Table 3). Mueck et al [13] used radiocarbon dating from the base of tall specimens of *C*. *australis* and *D*. *antarctica*, dividing height by age to give an average growth rate. Their findings were highly variable for *D*. *antarctica* with both the highest and lowest growth rates outside 2 standard deviations of our result. Mueck et al [13] concluded the higher end of their results for *D*. *antarctica* were probably overestimates. Ferwerda [22] measured tree fern frond spacing on coastal bluffs and new growth after fire in forests similar to our study. Despite the results of Ferwerda [22] being 1.8 standard deviations higher than our own, all of the post-fire measurements from that study were taken from tall specimens, for which our study indicates growth rates to be above average.

**Table 3:** Summary of tree fern growth studies in Victoria, south-eastern Australia.

Study	Location	Method	Species	Growth rate
Ferwerda (1981)	Western Port	Frond spacing	*C*. *australis*	105 mm/yr (n = 5)
Ferwerda (1981)	Ferntree Gully NP	Regrowth after fire	*C*. *australis*	113 mm/yr (n = 6)
Mueck et al (1996)	Toolangi	Radio carbon dating	*C*. *australis*	22–38 mm/yr (n = 2)
Mueck et al (1996)	Toolangi	Radio carbon dating	*D*. *antarctica*	5–88 mm/yr (n = 6)
This study	Central Highlands	Regrowth after fire	*C*. *australis*	73(+/-22) mm/yr (n = 163)
This study	Central Highlands	Regrowth after fire	*D*. *antarctica*	33(+/-13) mm/yr (n = 172)

### Environmental variables

We found only limited effects of environmental variables on growth rates, despite at the outset of the study predicting all three variables (elevation, slope and aspect) would be important based on results from other studies [6, 39]. Our analyses revealed that elevation had a very limited effect, with growth rates increasing marginally with increased elevation. This may indicate that temperature and rainfall, both of which are highly correlated with elevation in our study area, did not have an effect, or that there were additional factors that masked these effects. A lack of effect was unexpected due to temperature, in particular, having been shown to alter quantum yield [20, 25]. In addition, both variables have been identified as important determinants of the distribution of *C*. *australis* and *D*. *antarctica* [6, 40, 41]. It therefore appears that what affects the distribution of tree ferns may not influence growth rate.

### Geographic location

We did not measure sufficient or appropriate additional variables to determine why the growth rate of tree ferns would vary between different geographic locations. Different local factors are important as Toolangi State Forest was the area of fastest growth for *C*. *australis*, but slowest growth for *D*. *antarctica*. Wallaby Creek water catchment seemed very favourable to both species with *D*. *antarctica* growing significantly more rapidly relative to other locations. In New Zealand, Brock et al [6] found tree fern growth increased with decreasing latitude. In contrast, the geographic range of our study was far more restricted, but also spread more east-west with minimal difference in latitude. As such, latitude was unlikely to be a factor affecting our results.

### *Cyathea* grow faster than *Dicksonia*

We confirmed that *C*. *australis* grows faster than *D*. *antarctica*. This is perhaps not surprising given *Cyathea* as a genus has many very fast growing species and, on average, have larger crown spread than *Dicksonia* [16].*This* may in part be due to *C*. *australis* having a greater specific leaf area of 10.4m^2^/kg compared to 8.6m^2^/kg for *D*. *antarctica* [20] which provides for greater photosynthetic capability. Physiologically, *Cyathea australis* also has leaf hairs that may reduce UV-B exposure in the post-fire high growth irradiance environment. It is thought these leaf hairs also may explain why *C*. *australis* has a broader climatic niche that *D*. *antarctica* [20, 24]. Interspecific differences in growth rates also may be due to differences in rooting structure or due to resources extracted by bryophytes and epiphytes, which are in greater numbers on the moister fibrous trunks of *D*. *antarctica* than on the drier stipe shielded trunks of *C*. *australis* [42]. The difference in microclimatic conditions of locations where these tree fern species are found also may effect growth rates.

### Taller ferns grew more

Contrary to our expectations at the outset of this investigation of uniform growth rates for ferns of different height, we found that taller *C*. *australis* and *D*. *antarctica* had grown more than shorter individuals during the 5 year period of measurement. We considered four possible reasons for this: 1) The size and spread of the rosette of fronds, which increase in diameter as ferns become established; 2) shorter ferns being more negatively affected by the fire and being less capable of using fire released nutrients; 3) growth rates for these species were exponential; and 4) following increases in light availability due to overstorey canopy removal by fire, taller ferns being exposed to these increased light levels for longer as shorter ferns are over taken and shaded by regrowing tree species for a greater proportion of the study period. We examine these four explanations in detail below.

Our first proposition suggested the larger rosettes of older ferns may allow for greater growth due to increased capture of light (and therefore photosynthetic capacity) and re-direction of rainfall compared to shorter ferns with smaller crowns. Interception of rainfall by tall tree ferns may further limit rainfall reaching short individuals when they are situated beneath taller ones. New fronds pointing upwards funnel water into the top of the trunk where aerial roots surround the fronds, as well as fronds themselves being capable of direct uptake of moisture [12]. However, higher growth rates achieved through the effect of greater interception of both light and rainfall should taper once maximum crown diameter is reached, which from our observations, occurs when ferns are approximately one metre in height.

Given we measured growth following a major wildfire, the second possible explanation for our observation of taller ferns growing faster than short tree ferns, was the change in photosynthetic capability due to loss of fronds, potentially counter-balanced by a positive effect of increased growth due to increased availability of nutrients in the ash bed following fire [43]. Physical damage from fire could have greater effects on short ferns which have their crown closer to ground level and where fuel loads are generally highest. If fire damage was a factor affecting growth rates, we would expect aspect and slope to have been important covariates in our models because they have a strong influence on fire severity [44]. It also seems unlikely such effects would be influential beyond the first year unless the fire delayed post-fire re-sprouting in shorter ferns. We checked for such effects by viewing several hundred post-fire photographs of burned stands but found no evidence of retarded resprouting of short ferns compared to tall ones. Increased nutrients may be a factor underpinning changes in growth rates pre and post fire. However, as all our measurements were post-fire, this does not explain the greater rates of growth among taller ferns, unless dominant individuals were able to monopolize resources, which seems unlikely.

A third possible explanation for our results was that we had measured exponential growth, or that taller tree ferns inherently grew faster. Given that initial height of the tree ferns at the time of the 2009 fire determined how much they grew, and that this apparent increased growth rate did not seem to taper over the duration of our investigation (Figs 2 and 3), a possible conclusion was that we had discovered exponential growth throughout a plant’s life, a very unusual pattern for any plant species. The only example in the literature for tree ferns that suggested this may be possible was a Jamaican study on *Cyathea pubscens* that found stipe interval increased with trunk height, indicating possible growth acceleration with time [26], although this was not directly measured. To test this explanation, we modelled exponential equations from our data. However, given the longevity of these two species, our equations provided highly unrealistic results for old tree ferns with heights over 50 metres tall, suggesting another explanation was needed.

A fourth possible explanation for our results was canopy removal by fire of the surrounding trees and shrubs and subsequent sequential shading by dense regeneration. In the forests of the Central Highlands, following a high severity fire such as the one at the start of our study period, all strata have foliage removed [45] leading to greatly increased light availability. This is followed by a pulse of vigorous regrowth of eucalypts and *Acacia* germinating from seed. From other unpublished vegetation data we collected on post fire regrowth, measured across the same period as our study, we know the growth rate of this dense cohort of trees is rapid but is relatively uniform [46]. After a year to establish, the regrowth grows at approximately 1–1.5m/year. The regrowth is very dense and provides almost complete shade to plants beneath the canopy. The loss of overstorey canopy shading due to the fires created a period of greatly increased light penetration reaching the tree ferns, which may have led to an accelerated growth rate [6, 47]. For the shortest ferns in our study, the period of increased light availability due to absence of overstorey canopy may have lasted less than 12 months, while the tallest ferns studied would have had such conditions for three to five years, giving them more favourable growing conditions for longer.

Differential growth of individuals in sun, compared to those located in shade, has been observed in tree fern studies in Columbia [48]. Observations from New Zealand found different tree fern species occupy niches along a shade-light spectrum where growth varied, but importantly, where recruitment was also effected by shade levels [49]. A series of studies on the physiology of both *D*. *antarctica* and *C*. *australis*, in greenhouse and field conditions, showed high irradiance caused decreases in photosynthetic capacity [20, 21, 25]. However, these same studies also showed varying ability of these species to acclimatise to such changes in irradiance over periods up to 3 months. The one field-based study of the three, which was the only one studying tree ferns more than a year old, found the ability for seasonal acclimation [20]. Lower temperatures in winter were shown to reduce photosynthetic capacity, so it may be that the additional warmth of full sun exposure during winter allowed additional growth in our study despite our study finding temperature not to be influential. Actual growth rates were not measured in these studies. The results from Ferwerda [22] would support our concept of variable growth rates in tree ferns as it was the tallest ferns burnt by fire (and therefore exposed to more light for longer) that had the greatest growth rates in that study.

If tree ferns grow faster with increased available light, this may assist interpretation of the results of other studies. For example, a study in Costa Rica which concluded growth rates were related to whether the surrounding habitat was primary or secondary forest [50], only briefly mentioned light levels. Our study would suggest that increased light levels may have been important, with tree fern growth in open secondary forest being up to three times that of closed primary forest. The concept of greater growth rates in sun compared to shade was found to hold true in a study from the Andes, where *Cyathea caracasana* grew more rapidly when exposed to full sun compared to closed forest [27]. The conclusion was that *C*. *caracasana* was a species that was able to take advantage of gaps in the forest to grow rapidly and produce large volumes of spores, then slow down again but persist as shading increased. It would appear that tree ferns in Australia may follow a similar ‘sit and wait’ life strategy. However, our findings showing an increase in growth rate as ferns became taller was in contrast to a study of five *Cyathea* species in Costa Rica where growth remained constant over time [50], or in Japan where growth of *Cyathea spinulosa* slowed gradually over time [29]. It was also counter to the assumption (without evidence) made by Ferwerda [22] that growth rates of *C*. *australis* would slow as the ferns increased in height.

We do not know if tree ferns in Australia produce additional spores during times of increased light exposure and accelerated growth, but if this is the case, it could have significant implications for forest management if an aim is to encourage tree fern recruitment in areas where they have been reduced in number (such as logged stands or areas where tree fern harvesting occurs). In New Zealand, tree ferns rapidly colonise open areas with suitable conditions, however this does not appear to be the case in Australia [6, 51]. Improved recruitment of tree ferns across logged areas may be possible by retaining them around the edge of cutblocks, in undisturbed islands of forest retained across harvested areas where they are more likely to survive and where possible, within the cut area. By retaining tree ferns in multiple directions, this is likely to enhance post-disturbance recruitment because the tree fern’s spores are wind dispersed.

We did not specifically measure light interception, canopy cover or spore production by tree ferns. Further research measuring tree ferns in areas subject to different light regimes is required. Given *D*. *antarctica* tends to grow to a height of ~ 5-6m before collapsing, it should be possible to test whether it is age or height (and therefore access to light) that most strongly influences growth rate. If old fallen tree ferns grow at the same rate as much younger ferns which are the same height, this would lend weight to light being significant driver of growth rates. If however, they continue to grow at similar rates to those of the same age, it would indicate age is a more important driver of growth rates.

## Conclusions

Tree ferns are an important element of stand structure and species composition of many forests worldwide. They have numerous key ecological roles such as providing food, nesting sites and movement pathways for animals and being host sites for a wide diversity of epiphytic plants. Tree ferns are long lived and are commonly found in old growth forests. The factors that determine where tree ferns persist appear to be different from those that determine growth rates. Accurate estimation of their age remains difficult due to growth rates varying through a plant’s life, preventing the development of simple equations such as those that divide height by growth rate. Given our measurements were taken across a period when increased growth rates due to the increased light availability seems likely, the average growth rate for the two species of tree ferns are likely to be above longer term averages when greater shading is the norm. Despite being well adapted to fire, tree ferns are highly susceptible to logging [17, 18] and if spore production in these species is found to increase with increases in available light, this may have important implications for forest management. Our research, while a relatively short term cross-sectional study of these long-lived organisms, increases our understanding of their ability to grow rapidly following fire and may assist with the management of these important species.

## Supporting information

S1 Table Summary of locations, number of ferns and variables measured. (DOCX)

S2 Table Modelling results for the two fern species. Where L-95% CI and U-95% CI are the lower and upper end points of the 95% credible interval and Btail is a measure of support (see Methods for more detail).
(DOCX)
