# Supplementary material for: Non-linear growth in tree ferns, Dicksonia antarctica and Cyathea australis
Source: PLoS One. 2017 May 11;12(5):e0176908. doi: 10.1371/journal.pone.0176908 (PMC5426625; doi:10.1371/journal.pone.0176908)
Supplement: S1 Table — (DOCX) [file pone.0176908.s001.docx]

**BLAIR et al – Non-linear growth of tree ferns, *Dicksonia antarctica* and *Cyathea australis***

**Reference: PONE-D-16-31845R2**

**S1 Table. Summary of locations, number of ferns and variables measured.**

| **Geographic Region** | **Location** | **# ferns measured** | | **Aspect** | **Altitude** |
| --- | --- | --- | --- | --- | --- |
|  |  | **Cyathea** | **Dicksonia** |  |  |
| Marysville | Yellow dog | 10 |  | S | 500 |
|  | Yellow dog + 1k | 10 | 10 | S | 605 |
|  | Olsens |  | 10 | SW | 840 |
|  | Lady Talbot bottom | 10 | 2 | flat | 395 |
|  | Tommys Bend |  | 10 | W | 985 |
|  | Tommys Bend/Yellow dog | 1 | 10 | W | 975 |
|  | Site 805 | 10 | 10 | N | 924 |
|  | Paradise Plains Rd, 1.8km below Keppel Lookout | 10 |  | N | 640 |
| O'Shannassy | Poley/Paradise Plains |  | 10 | SW | 940 |
|  | Rd 9 | 1 | 10 | flat | 860 |
|  | Rd 9 / 7 |  | 10 | W | 945 |
|  | Site 512 | 1 | 10 | flat | 770 |
|  | Rd 9 1km from Rd 7 |  | 10 | flat | 830 |
| Toolangi | Nolans | 10 | 10 | flat | 690 |
|  | Chum Creek -5km | 10 |  | SW | 275 |
|  | Chum Creek -3km | 10 |  | SW | 355 |
|  | Blowhard at 277 | 10 |  | NW | 640 |
|  | Starlight Rd | 10 | 10 | flat/E | 715 |
|  | Klondyke/Mt Klondyke | 10 | 10 | NE | 682 |
| Wallaby Creek | Rd 3, 200m from Rd 10 | 10 |  | flat/S | 530 |
|  | Rd 3, 1km from Rd 10 | 10 |  | S | 590 |
|  | Rd 3, 0.5km W of Rd 15 | 10 | 10 | S | 670 |
|  | Rd 3, 0.7km S of Rd 12 | 10 | 10 | S | 685 |
|  | Rd 3 / Rd 14 intersection | 10 | 10 | flat | 690 |
|  | Rd 14, 0.2km E of Rd 2 at the creek |  | 10 | flat/NE | 660 |
