# Supplementary material for: Non-linear growth in tree ferns, Dicksonia antarctica and Cyathea australis
Source: PLoS One. 2017 May 11;12(5):e0176908. doi: 10.1371/journal.pone.0176908 (PMC5426625; doi:10.1371/journal.pone.0176908)
Supplement: S2 Table — Where L-95% CI and U-95% CI are the lower and upper end points of the 95% credible interval and Btail is a measure of support (see Methods for more detail). (DOCX) [file pone.0176908.s002.docx]

**BLAIR et al – Non-linear growth of tree ferns, *Dicksonia antarctica* and *Cyathea australis***

**Reference: PONE-D-16-31845R2**

**S2 Table. Modelling results for the two fern species. Where L-95% CI and U-95% CI are the lower and upper end points of the 95% credible interval and Btail is a measure of support (see Methods for more detail).**

*Cyathea australis*

|  | Posterior Mean | L-95% CI | U-95% CI | Btail |
| --- | --- | --- | --- | --- |
| GR: Marysville, Aspect: North | 37.923 | 15.766 | 62.391 | **0.002** |
| GR:Toolangi vs Marysville | 13.856 | 5.630 | 22.474 | **0.002** |
| GR: Wallaby Creek vs Marysville | 11.686 | 2.055 | 21.191 | **0.009** |
| Elevation | 0.019 | -0.006 | 0.044 | 0.066 |
| Slope | -0.016 | -0.443 | 0.404 | 0.460 |
| Aspect: Other | 5.502 | -4.142 | 15.134 | 0.126 |
| Initial Height (m) | 4.902 | 2.015 | 7.696 | **<0.001** |
| GR: Toolangi vs Wallaby Creek | 2.17 | -8.914 | 12.660 | 0.343 |

GR = Geographic Region

*Dicksonia antarctica*

|  | Posterior Mean | L-95% CI | U-95% CI | Btail |
| --- | --- | --- | --- | --- |
| GR: Marysville, Aspect: North | 3.662 | -16.762 | 24.794 | 0.360 |
| GR: O'Shan vs Marysville | 3.745 | -2.374 | 9.95 | 0.118 |
| GR:Toolangi vs Marysville | 2.261 | -4.799 | 9.559 | 0.273 |
| GR: Wallaby Creek vs Marysville | 12.514 | 5.934 | 20.201 | **0.002** |
| Elevation | 0.016 | -0.005 | 0.036 | 0.061 |
| Slope | 0.333 | 0.069 | 0.637 | **0.010** |
| Aspect: Other | -0.195 | -5.273 | 5.105 | 0.475 |
| Initial Height (m) | 6.000 | 3.188 | 8.889 | **<0.001** |
| GR: O’Shan vs Toolangi | 1.484 | -6.333 | 9.329 | 0.357 |
| GR: O’Shan vs Wallaby Creek | -8.769 | -15.393 | -2.305 | **0.004** |
| GR: Toolangi vs Wallaby Creek | -10.253 | -16.241 | -3.855 | **0.002** |

GR = Geographic Region
